# Altered calcium influx of peripheral Th2 cells in pediatric Crohn's disease: infliximab may normalize activation patterns

**DOI:** 10.18632/oncotarget.10036

**Published:** 2016-06-14

**Authors:** Csaba Orbán, Dolóresz Szabó, Anna Bajnok, Barna Vásárhelyi, Tivadar Tulassay, András Arató, Gábor Veres, Gergely Toldi

**Affiliations:** ^1^ First Department of Pediatrics, Semmelweis University, Budapest, Hungary; ^2^ Department of Dietetics and Nutrition Sciences, Faculty of Health Sciences, Semmelweis University, Budapest, Hungary; ^3^ Department of Laboratory Medicine, Semmelweis University, Budapest, Hungary; ^4^ MTA-SE, Pediatrics and Nephrology Research Group, Budapest, Hungary

**Keywords:** Th2, Kv1.3, IKCa1, infliximab, Crohn's disease, Immunology and Microbiology Section, Immune response, Immunity

## Abstract

**Objective:**

Crohn's disease is a chronic inflammation of the gastrointestinal tract with an abnormal immune phenotype. We investigated how intracellular calcium kinetics of Th1 and Th2 lymphocytes alter upon specific inhibition of Kv1.3 and IKCa1 channels in pediatric Crohn's disease.

**Study design:**

Blood was taken from 12 healthy and 29 Crohn's disease children. Of those, 6 were switched to infliximab and re-sampled after the 4th infliximab treatment. Intracellular calcium levels were monitored using flow cytometry in the presence or absence of specific inhibitors of Kv1.3 and IKCa1 potassium channels.

**Results:**

In Crohn's disease treated with standard therapy, calcium response during activation was higher than normal in Th2 cells. This was normalized *in vitro* by inhibition of Kv1.3 or IKCa1 potassium channels. After the switch to infliximab, potassium channel function and expression in Th2 lymphocytes were comparable to those in Th1 cells.

**Conclusion:**

These results may indicate that potassium channels are potential immune modulatory targets in Crohn's disease.

## INTRODUCTION

Crohn's disease (CD) is a chronic inflammatory disease of the bowels [1]. Immune dysfunction, particularly that of adaptive immunity leading to a pro-inflammatory status is a characteristic hallmark and an important contributing factor in CD [2]. Locally, in the inflamed lamina propria the number of pro-inflammatory Th1 and Th17 cells is elevated [3, 4], while that of anti-inflammatory Treg cells is decreased.

In addition to the altered immune phenotype, short-term activation characteristics of T cells may be also abnormal in CD [5, 6]. This is represented by changes in calcium handling during T cell activation. The biphasic calcium signal that occurs following the T cell receptor stimulation is an essential part of the activation process evoked by antigen binding to the T cell receptor. During the first part of activation, the intracellular stores extrude calcium into the cytosolic compartment, then the depletion of the endoplasmic reticulum (ER) evokes the calcium influx from the extracellular space *via* store operated calcium channels (SOCE) [7]. To maintain the negative membrane potential, and to sustain further calcium entry into the cells, potassium ions efflux from the cells through the voltage gated Kv1.3 and calcium sensitive IKCa1 potassium channels [8].

Our research group had investigated intracellular calcium kinetics during stimulation of different T cell subsets with a specific novel flow cytometry method. This method is able to describe the kinetic change that occurs during the measurement by fitting different functions to the kinetic parameter values [9]. Different parameters of these functions are comparable in an objective manner. With the use of this method, we performed measurements in different age groups [10] and pathological conditions. We have demonstrated distinct calcium handling profiles of Th1, Th2, Th17 and Treg cells in healthy adults [11]. In addition, we also reported that autoimmune disorders have a major impact on T cell calcium homeostasis. During the short-term activation of peripheral T cells in patients with rheumatoid arthritis (RA) [12], multiple sclerosis [13] and type 1 diabetes [14], we found altered intracellular calcium kinetics, that were, at least partly, attributed to altered function and/or expression of lymphocyte potassium channels. We also demonstrated that some immune modulatory therapies, also used in CD, may have an impact on T cell function. Indeed, biological agents including infliximab (IFX) normalized calcium response during short term stimulation of peripheral T cells in RA [15].

In this study we investigated calcium handling of Th1 and Th2 cells and the function of major lymphocyte potassium channels in pediatric CD patients treated with different therapeutic regimes.

Based on earlier results in other autoimmune conditions, we hypothesized that lymphocyte calcium influx kinetics may be altered in pediatric CD. We also hypothesized that different groups created by disease severity would show distinct lymphocyte potassium channel expression and sensitivity to selective blockers.

## RESULTS

### Laboratory parameters

Laboratory parameters (Table 1) indicate that neutrophil percentages were significantly higher in CD patients compared to healthy children, while lymphocyte percentages were decreased. C-reactive protein (CRP) values were higher in the moderate-severe group before IFX therapy, but not significantly different from the control and conventional groups. IFX therapy decreased Pediatric Crohn's Disease Activity Index (PCDAI) scores as well as the elevated CRP levels.

**Table 1:** Clinical parameters of the investigated children

	Healthy children *n* = 12	Conventional therapy *n* = 23	Before 1st IFX *n* = 6	After 4th IFX *n* = 6
**Age (years)**	13 [10-16]	15 [9-17]	16 [14-17]	16 [14-17]
**Gender (boy/girl)**	5/7	9/14	3/3	3/3
**C- reactive protein (mg/L)**	2 [1-3]	6 [0-24]	31 [8.75-41.25]	1.50^c^ [0.25-5.75]
**White Blood Cell (G/L)**	6.50 [5.53-7.15]	6.70 [5.90-9.20]	9.70 [6.28-12.38]	6.00 [4.80-9.60]
**Neutrophill Granulocyte (% of White Blood Cells)**	49.90 [47.50-57.20]	61.40^a^ [58.60-66.60]	73.60^a^ [66.40-79.23]	69.30^a^ [65.18-74.63]
**Lymphocyte (% of White Blood Cells)**	38.50 [34.<70-43.70]	21.30^a^ [18.<20-28.90]	18.60^a^ [13.85-20.88]	20.25^a^ [16.65-26.10]
**Thrombocyte Platelet (G/L)**	374 [325-396]	401 [294-565]	465 [429-584]	342 [287-378]
**Pediatric Crohn's Disease Activity Index**	-	10 [5-35]	36^b^ [35-39]	0^c^ [0-7.5]

Data are presented as median [IQR]

a *p* < 0.05 *vs*. Healthy children

b *p* < 0.05 *vs*. Conventional therapy

c *p* < 0.05 *vs*. Before 1st IFX

### Immune phenotype

The prevalence of Th1 cells was higher than normal in CD (Table 2). In the moderate-severe disease group, before the 1^st^ IFX therapy, the percentage of Th2 cells increased in peripheral blood. After the 4^th^ IFX therapy, the ratio of Th1/Th2 further increased.

**Table 2:** Distribution of Th1 and Th2 cells in the different groups

	Healthy Children controlls	Conventionally treated CD	Moderate-severe CD Before 1^st^ IFX therapy	Moderate-severe CD After 4^th^ IFX therapy
Th1%/lymphocytes	10,73 [0,52-30,80]	17,90 [0,<32-49,34]	21,84 [2,10-39,27]	33,05 [2,01-43,89]
Th2%/lymphocytes	13,99 [0,91-31,60]	9,09 [0,42-49,35]	13,72 [1,16-23,68]	19,53 [3,22-44,50]
Th1%/Th2%	0,77	1,97	1,59	1,69

Data are presented as median [range].

### Calcium influx characteristics

**Figure 1:**
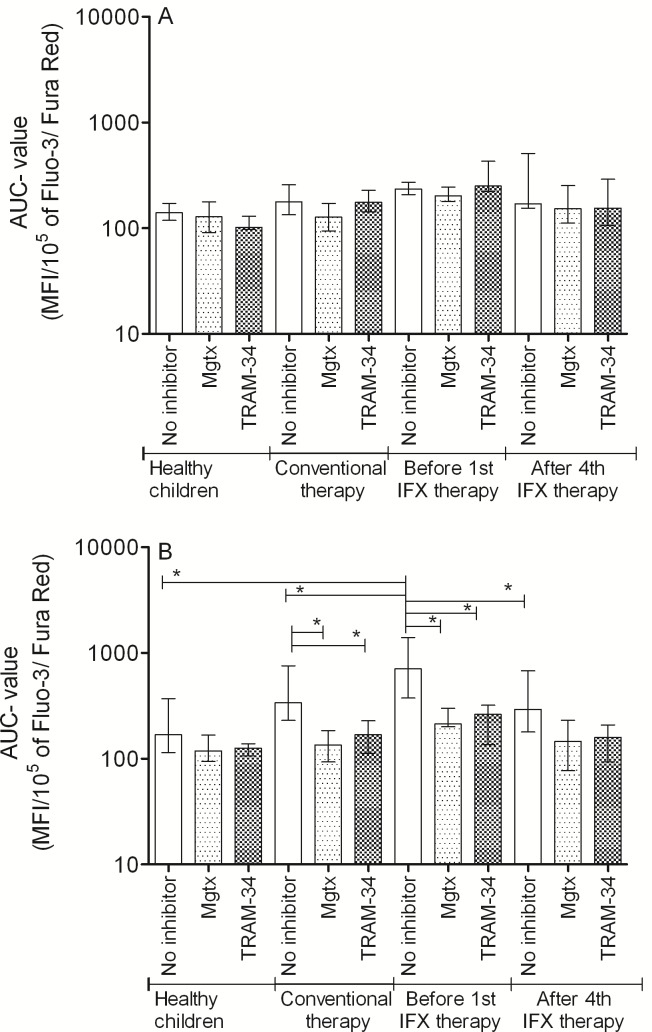
AUC parameter changes of the cytoplasmic calcium concentration altered by potassium channel inhibitors following PHA activation of A: Th1 and B: Th2 cells (median, IQR) *p < 0.05.

Our results show that in healthy children Th1 and Th2 cells have similar calcium influx characteristics upon activation (Table 3A). In CD, Th2 cells show intensified calcium influx compared to Th1 subpopulation. Although Start values were similar, the Maximum, and Area Under the Curve (AUC) values were significantly higher in Th2 cells. This elevated calcium signal was normalized in the presence of both margatoxin (MGTX) and triarylmethane (TRAM-34) potassium channel inhibitors (Figure 1). Potassium channel inhibitors only had an effect on the calcium homeostasis of Th2 cells, while that of the Th1 subset remained unaffected.

**Table 3A:** Calcium influx parameters and Kv1.3 channel expression data of healthy controls and conventionally treated CD patients

	Healthy Children					
	No inhibitor		Mgtx (4 nM)		TRAM-34 (240 nM)	
	Th1	Th2	Th1	Th2	Th1	Th2
Starting value (MFI/10^2^)	226 [136-332]	194 [125-281]	192 [106-299]	181 [108-301]	152 [133-194]	158 [137-197]
Maximum value (MFI/10^2^)	234 [139-364]	391 [134-2919]	225 [115-384]	214 [118-397]	196 [<171-241]	261 [193-265]
Slope at first 50% value	301 [1-10321]	3258 [105-175245]	24 [10-1726]	20 [4-479]	26 [22-37]	39 [14-57]
AUC (MFI/10^5^)	140 [95-201]	168 [77-563]	128 [66-183]	118 [68-186]	102 [97-130]	126 [106-140]
MFI Anti-Kv1.3	91 [<15-669]	160 [10-1194]				
	**Conventionally treated CD**					
	**No inhibitor**		**Mgtx (4 nM)**		**TRAM-34 (240 nM)**	
	**Th1**	**Th2**	**Th1**	**Th2**	**Th1**	**Th2**
Starting value (MFI/10^2^)	293 [120-854]	394 [145-1413]	193 [108-552]	216 [107-689]	232 [15-619]	223 [146-570]
Maximum value (MFI/10^2^)	340 [171-1099]	1094 [148-2588]^b^	229 [115-663]	246 [113-815]^a^	289 [31-754]	307 [191-782]^a^
Slope at first 50% value	69 [2-10262]	123 [5-19105]	97 [1-3126]	31 [1-5402]	174 [1-2491]	170 [13-30706]
AUC (MFI/10^5^)	178 [02-411]	338 [132-1054]^b^	127 [66-374]	135 [65-413]^a^	170 [9-414]	169 [97-431]^a^
MFI Anti-Kv1.3	179 [52-1191]	358 [67-1315]^b^				

Data are presented as median [IQR]

a *p* < 0.05 *vs*. no inhibitor (control) treatment in same disease group, same cell subset

b *p* < 0.05 *vs*. Th1 cell values in same disease group, same inhibitor treatment

c *p* < 0.05 *vs*. Healthy children in same cell subset, same inhibitor treatment

e *p* < 0.05 *vs*. Conventional therapy in same cell subset, same inhibitor treatment

f *p* < 0.05 *vs*. Before IFX therapy in same cell subset, same inhibitor treatment

In the moderate-severe disease group, before the 1^st^ IFX therapy, Th2 predominance was observed compared to the results of healthy controls and classically treated patients (Figure 1). This predominance can be observed not only in Maximum and AUC values, but in the Start values as well. Th1 cells of the moderate-severe disease group did not show altered calcium influx following activation compared to the other groups (Table 3B). The elevated calcium influx characteristics of Th2 cells were normalized by both MGTX and TRAM-34.

**Table 3B:** Calcium influx parameters and Kv1.3 channel expression data of the moderate-severe CD group

	Before 1st IFX therapy					
	No inhibitor		Mgtx (4 nM)		TRAM-34 (240 nM)	
	Th1	Th2	Th1	Th2	Th1	Th2
Starting value (MFI/10^2^)	400 [330-566]	1023 [100-2597]^b^,^c^,^e^	329 [257-493]	340 [272-614]^a^	399 [2-669]	432 [1-617]^a^
Maximum value (MFI/10^2^)	436 [393-583]	2233 [1176-2621]^b^,^c^,^e^	393 [361-550]	387 [364-798]^a^	407 [2-805]	455 [10-794]^a^
Slope at first 50% value	2665 [81-21390]	8531 [156-129330]^e^	90 [30-4543]	59 [25-10837]^a^	710 [23-4167]	3187 [1-7725]^a^
AUC (MFI/10^5^)	236 [199-283]	709 [279-1572]^b^,^c^,^e^	203 [157-296]	213 [169-328]^a^	241 [1-513]	263 [5-375]^a^
MFI Anti-Kv1.3	165 [87-1230]	327 [86-413]				
	**After 4th IFX therapy**					
	**No inhibitor**		**Mgtx (4 nM)**		**TRAM-34 (240 nM)**	
	**Th1**	**Th2**	**Th1**	**Th2**	**Th1**	**Th2**
Starting value (MFI/10^2^)	260 [127-495]	546 [216-1136]^f^	237 [162-402]	247 [162-390]	254 [74-496]	266 [77-712]
Maximum value (MFI/10^2^)	276 [154-315]	904 [306-1813]^f^	213 [88-320]	244 [88-551]	260 [91-512]	292 [90-231]
Slope at first 50% value	81 [2-10178]	195 [21-1146]^f^	30 [8-63]	31 [6-81]	1276 [32-3517]	908 [21-17080]
AUC (MFI/10^5^)	155 [83-187]	293 [170-894]^b^,^f^	153 [105-280]	146 [50-298]	155 [51-298]^b^	159 [51-242]
MFI Anti-Kv1.3	122 [49-983]	99 [48-178]				

Data are presented as median [IQR]

a *p* < 0.05 *vs*. no inhibitor (control) treatment in same disease group, same cell subset

b *p* < 0.05 *vs*. Th1 cell values in same disease group, same inhibitor treatment

c *p* < 0.05 *vs*. Healthy children in same cell subset, same inhibitor treatment

e *p* < 0.05 *vs*. Conventional therapy in same cell subset, same inhibitor treatment

f *p* < 0.05 *vs*. Before IFX therapy in same cell subset, same inhibitor treatment

IFX therapy evoked a massive effect on calcium homeostasis of Th2 cells, reflected by the normalization of Start, Maximum, AUC, and even Slope values. MGTX and TRAM-34 did not evoke further decrease on calcium levels in the IFX treated group.

### Kv1.3 potassium channel expression

In healthy children, the voltage gated Kv1.3 potassium channel expression was comparable in Th1 and Th2 subsets. In CD patients, Th2 cells express Kv1.3 potassium channel at a higher rate. In moderate-severe patients before the 1^st^ IFX therapy, Th2 cells showed higher median value of expression than Th1 cells. After the 4^th^ IFX therapy, this difference decreased to nearly equal levels.

**Figure 2:**
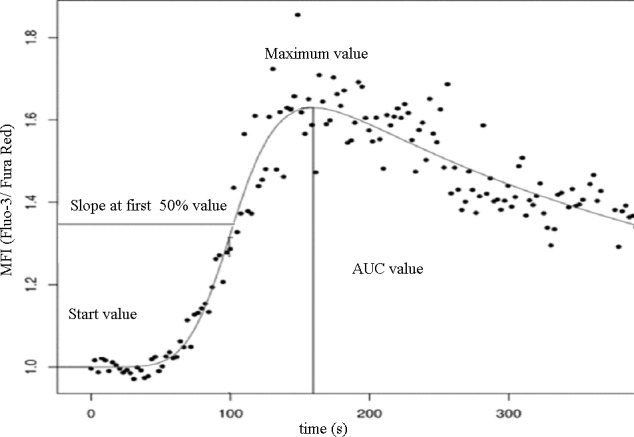
Investigated parameters of the double logistic function of cytoplasmic calcium concentration

## DISCUSSION

In this study we investigated routine laboratory parameters, distribution and calcium influx characteristics of Th1 and Th2 cell subpopulations, and their Kv1.3 potassium channel expression in classically and IFX treated pediatric CD patients.

Routine laboratory parameters indicated higher neutrophil and lower lymphocyte percentages in the CD groups. CRP values were also elevated in the moderate-severe group before IFX treatment and were restored to normal levels by IFX. These findings are in line with earlier results [16]. PCDAI scores were above 30 in the moderate-severe group before IFX therapy. The efficacy of IFX therapy is indicated by a notable decrease in PCDAI scores.

Interestingly, phenotypic alterations indicated higher percentages of Th1 within the lymphocyte population in classically treated CD. This elevated prevalence of Th1 cells in the peripheral blood further increased in the moderate-severe group before the 1^st^ IFX therapy. IFX treatment did not decrease the percentages of Th1 cells in the peripheral blood, but the prevalence of Th2 cells was increased [4]. However, since lymphocyte counts are higher in healthy controls, the absolute number of Th1 cells is only higher in the moderate to severe CD group. Likewise, the absolute number of Th2 cells is lower in CD patients than normal and decreased after infliximab treatment due to the lower lymphocyte counts in these patients following infliximab therapy. The migration of Th1 cells into the inflamed lamina proparia from the periphery might also play a role in this phenomenon. This presumption needs to be verified by further investigations in the future.

Our results on calcium influx kinetics clearly indicated a distinct profile of classically treated CD from age-matched healthy individuals. This is similar to previous findings of our team in autoimmune disorders like rheumatoid arthritis and type 1 diabetes [12, 14], which were also characterized by a significant alteration of T cell calcium influx kinetics.

Th2 cells in the conventionally treated group and in moderate-severe patients before IFX treatment showed higher initial and peak cytoplasmic calcium concentrations as well as AUC values, hence this may indicate that this subpopulation is more active during early phase signaling compared to Th1 cells. At the first glance, this is in contrast with findings describing that CD is a Th1 type disorder [17, 18]. The difference may come from the fact that most of the studies were performed on adult patients and describe the local cytokine and cellular milieu in the gut. Instead, studies that investigated pediatric subjects also found a shift toward Th2 populations in the peripheral blood [19]. This shift observed in the bloodstream might partially be due to the extravasation of Th1 cells in the gut, however, it is not confirmed by prevalence values of the current study. While the conclusion of others was based on prevalence data of the distinct T cell subsets, our results give further information about the function of these cells.

One of our most interesting observation comes from the measurements performed on the group where blood samples were obtained before the first and after the 4^th^ IFX therapy on patients with an initial PCDAI score > 30. Paired sample analysis of this group indicated that IFX has a clear effect on calcium influx characteristics of Th2 cells. This effect has never been mentioned before in the literature, and it is unclear which signaling pathway is responsible for the observed phenomenon. We hypothesize that the chimeric antibody may alter the surface expression of calcium (e.g. SOCE, Cav1) or potassium channels (IKCa1, Kv1.3) on lymphocytes, as it is a high molecular weight protein, hence it cannot penetrate the cell membrane and alter other intracellular channels that are crucial to calcium homeostasis of the cells, such as the sarco/endoplasmic reticulum calcium ATPase (SERCA), or ryanodine receptors [20]. The identification of the exact signaling pathways requires further specific studies aimed at detecting the affected messenger molecules.

The investigated potassium channels have a crucial role in the altered early phase T cell activation in pediatric CD. Application of selective blockers of the IKCa1 and Kv1.3 potassium channels is a possible new target for autoimmune therapy [21], and in a colitis murine model they already proved to be effective and fairly selective for the over-activated cell types [22]. In our study we could justify that both MGTX and TRAM has the potential to lower the increased calcium influx in Th2 cells nearly to the level observed in healthy individuals in case of the conventionally treated group. This effect may be due to the higher expression of Kv1.3 potassium channels on Th2 cells. This result is in line with other studies showing that the expression of Kv1.3 was elevated on the surface of CD4+ cells in ulcerative colitis, and levels of Kv1.3 correlate well with the higher expression of TNF-α, and may serve as a good marker of disease activity [21]. Hansen et al. also found Kv1.3 and IKCa1 potassium channels to be to be important in ulcerative colitis and declared them as possibly relevant pharmacological targets [5]. Furthermore, our results on potassium channel inhibition are similarly promising as the findings of Di Sabatino et al., who found the calcium release activated (CRAC) channel inhibition on lamina propria mononuclear cells (LPMC) may have beneficial therapeutic effects [23].

We also investigated the effect of lymphocyte potassium channel inhibition on the samples of moderate-severe, IFX treated subjects. We found that the decrease in intracellular calcium concentration induced by IFX cannot be further enhanced by the application of MGTX or TRAM. This seems to be logical, since the calcium homeostasis of T cells is regulated precisely [20], and a basal level of calcium is necessary for the proper function of resting lymphocytes, thus controlling biochemical mechanisms do not allow an extreme degree of alteration. Based on our results, the future potential of lymphocyte potassium channel inhibitors might be as simultaneous therapeutic agents besides IFX therapy, to lower the therapeutic dose of IFX. This approach might the possibility of decreasing both the cost and side-effects of IFX treatment.

Interestingly, our current results proved to be fairly selective in modulation of the abnormal calcium influx of peripheral Th2 cells, while our pervious studies show only moderate selectivity on CD8 lymphocytes in multiple sclerosis or on Th1 cells in type 1 diabetes mellitus [13, 14].

A limitation of our study is the fact that measurements were executed on peripheral blood samples, which may not reflect the circumstances in the inflamed mucosa. Further studies are needed to investigate whether our findings correspond to alterations in tissue samples. Another limitation is the small number patients who received IFX treatment. Specific signal pathway analysis and larger clinical trials will help support our conclusions.

Keeping in mind the above limitations, we conclude that the observed functional Th2 predominance in peripheral blood of pediatric CD patients might be shifted to normal values by inhibitors of Kv1.3 and IKCa1 lymphocyte potassium channels. IFX therapy may also reduce the elevated activation of the Th2 subset in patients with severe symptoms. The potential simultaneous application of these agents with IFX require further studies, as they appear to be promising novel therapeutic alternatives or complements of IFX therapy. Further investigations performed on gut biopsy samples are also needed to confirm the relevance of our findings.

## MATERIALS AND METHODS

### Sample collection

Peripheral blood samples were taken from 12 healthy children (5 boys and 7 girls; age: 13 (10-16) years, median (interquartile range)), and 23 conventionally treated CD patients diagnosed according to the Porto criteria system [24], and classified by the PCDAI scale [25] (9 boys and 14 girls; age: 15 years (9-17), median (interquartile range)). These patients received azathioprine and 5-acetylsalicylic acid (5-ASA) or mesalazine without systemic steroids. 6 patients (3 boys and 3 girls; age: 16 years (14-17), median (interquartile range)) with a PCDAI score > 30 who were switched to IFX (5mg/kg) were selected for measurements before the first, and after the 4th IFX-treatment. The distribution of the diseased intestines was homogenous in all patients with no specific predominant localization of the disease. The following laboratory parameters were also determined using routine laboratory procedures: CRP, White Blood Cell (WBC), neutrophil, lymphocyte and thrombocyte percentages. Informed consent was obtained from parents of all subjects, and our study was reviewed and approved by an independent ethical committee of the institution. The study was adhered to the tenets of the most recent revision of the Declaration of Helsinki.

### Calcium influx measurements

Peripheral blood mononuclear cells (PBMCs) were separated by a standard density gradient centrifugation (25 min, 400 g, 22°C) from freshly drawn blood. This cell suspension was washed twice in PBS. From then on, cells were kept throughout staining with fluorescent markers, treatment with inhibitors and measurement on flow cytometer in a modified RPMI-1640 medium. The calcium concentration of this medium was set to 2 mM by addition of crystalline CaCl_2_.

PBMCs were incubated with the following anti-human mAbs: anti-CD4 PE-Cy7 (clone SK3, BioLegend, San Diego, CA, USA), anti-CXCR3 APC (clone 1C6/CXCR3, BD Biosciences, San Jose, CA, USA), anti-CCR4 PE (clone 1G1, BD Biosciences) as well as anti-Kv1.3 FITC (Sigma-Aldrich, St. Louis, MO, USA) according to the manufacturers’ instructions. Cytoplasmic free calcium levels were detected by loading the cells with the 1:2 ratio of Fluo-3-AM and Fura Red fluorescent dyes (Biotium, Hayward, CA, USA) and 0.02 % Pluronic F-127 (Molecular Probes, Carlsbad, CA, USA) for 20 minutes at 30°C. Cells were washed once before measurement.

PBMCs were divided into four vials with equal cell numbers. One vial was treated with MGTX (4 nM; Sigma-Aldrich), a selective blocker of the Kv1.3 channel, for 15 min before measurement. Another vial was treated with a triarylmethane compound, TRAM-34 (240 nM; Sigma-Aldrich), a specific inhibitor of the IKCa1 channel, for 10 min before measurement. The third vial was used as control. The fourth vial was incubated with 1 μg anti-Kv1.3 (Sigma-Aldrich) besides the mAbs used for surface staining. In all cases, samples were kept at 30°C in dry bath, until the measurements.

At the beginning of the kinetic measurements, a 2 min baseline of calcium level was recorded. T cell activation was initiated by phytohemagglutinin (PHA, 15 μg/ml final concentration) as described earlier. Fluorescence emission of sequentially measured cells was monitored for 10 minutes. Average cell acquisition rate was 1000 cells/s. In case of the Kv1.3 expression measurements, 500.000 cells were recorded, and the mean fluorescence intensity (MFI) values were calculated.

All measurements were performed on a BD FACSAria flow cytometer (BD Biosciences). Single-stained controls were applied to perform compensation.

### Data analysis

The population of lymphocytes was gated according to forward and side scatter characteristics. CD4+ CXCR3+ cells were regarded as Th1 cells, CD4+ CCR4+ cells were regarded as Th2 cells population.

Data acquired from the measurements were evaluated with specific software developed at our laboratory (FacsKin, available at: www.facskin.com). The core of this software is an algorithm [26], based on the calculation of double-logistic functions for each recording. The software also calculates parameter values describing each function, such as the start value, representing the initial cytoplasmic calcium concentration of resting cells, the maximum value, the slope parameter, describing the magnitude of increase during calcium influx, and the AUC value, corresponding to the sum cytoplasmic calcium increase (Figure 2).

Comparisons of the calculated parameters between T cell subpopulations in the same patient group as well as between patient groups within the same T cell subset were made with the Kruskall-Wallis test, while the effect of the applied channel blockers were calculated using the Wilcoxon-test, as Kolmogorov-Smirnoff analysis indicated non-normal distribution of data. Statistics were calculated at 5% significance level (*p* = 0.05) using the GraphPad Prism 5 software (La Jolla, CA, USA).
